# Neutrophil-to-lymphocyte ratio; platelet-to-lymphocyte ratio; systemic immune-inflammatory Index: inflammatory indicators of cognitive impairment in schizophrenia patients

**DOI:** 10.3389/fpsyt.2025.1552451

**Published:** 2025-04-11

**Authors:** Ke Chen, Lina Wang, Houmei Ning, Huiqing Pan, Weibo Zhang

**Affiliations:** ^1^ Pujiang Hospital of Shanghai Mental Health Center, Minhang District Mental Health Center of Shanghai, Shanghai, China; ^2^ Shanghai Mental Health Center, Shanghai Jiao Tong University School of Medicine, Shanghai, China

**Keywords:** cognitive impairment, neutrophil-to-lymphocyte ratio, platelet-tolymphocyte ratio, systemic immune-inflammatory index, schizophrenia

## Abstract

**Objective:**

The present study sought to evaluate the correlation between cognitive impairment (CI) and inflammatory indicators such as neutrophil-to-lymphocyte ratio (NLR), platelet-to-lymphocyte ratio (PLR), and systemic immune-inflammatory index (SII) in schizophrenia patients.

**Methods:**

This study included 331 schizophrenia inpatients. General data and laboratory findings (neutrophil, lymphocyte, platelet, etc.) were gathered, and then calculating NLR, PLR, and SII. A Chinese version of the Mini Mental State Examination (MMSE) was used for the assessment of cognitive function, and then the patients in the CI group were categorized into mild CI, moderate CI, and severe CI groups. Comparing the differences in NLR, PLR, and SII between the CN group and the CI group, as well as different CI groups, and analyzing the relationship between the NLR, PLR, and SII and the mechanism of CI in schizophrenia.

**Results:**

There were 145 (43.8%) patients with cognitive impairment. Compared to the CN group, the CI group had higher NLR, PLR, and SII than the CN group, although their lymphocyte was lower. The NLR and SII were higher in the moderate CI group than in the mild CI group. NLR, PLR, and SII were significantly inversely correlated with the total score of cognitive function and scores across all aspects, whereas lymphocytes were considerably positively correlated. Higher NLR, PLR, and SII were substantially related to an increased risk of CI, but higher lymphocytes were associated with a decreased risk of CI.

**Conclusion:**

NLR, PLR, and SII may be serum inflammatory markers of CI in schizophrenia, and lymphocytes may be protective variables for cognitive function in schizophrenia.

## 1 Introduction

The cause of schizophrenia, a severe mental condition, remains unknown. It impairs patients’ social functioning by causing anomalies in perception, emotion, will, conduct, and thought. Positive and negative symptoms were previously believed to be the most common forms of symptoms related to schizophrenia. In recent years, psychiatrists have increasingly focused on cognitive impairment (CI) as an independent third core symptom of schizophrenia, based on an increasing number of clinicalobservations and research findings. CI refers to visuospatial orientation, language, calculation, memory, executive, and comprehension judgment of several degrees of impairment (1–3), reflecting the patient’s function. Studies have shown CI has more severe effects on patients’ social functioning. Schizophrenia patients usually have worse cognitive function compared to the general population (4, 5). Even though a patient’s psychiatric symptoms have subsided, CI still exists, causing decreased treatment adherence and increased risk of relapse of the illness, which is closely linked to unfavorable outcomes (6). Therefore, it is important to find effective cognitive interventions to improve the prognosis of patients with schizophrenia. However, there are presently no reliable medications to treat CI in schizophrenic patients. Antipsychotic drugs could improve psychiatric symptoms but do not improve cognitive function (7). We may find more effective ways to improve patients’ cognitive function if we have a deeper understanding of the biological mechanism of cognitive CI in schizophrenia patients. However, the pathophysiology of CI in schizophrenia is yet unknown.

Schizophrenia, a complex psychiatric disorder with unclear etiology, involves multi-system interactions in its pathophysiology. Immune dysregulation has been consistently observed in schizophrenia patients, with chronic inflammation implicated in disease mechanisms (8). Ballaz and Bourin et al. (9) emphasized the potential critical role of neuroinflammation in schizophrenia pathogenesis. They proposed that microglial activation, cytokine imbalance, hypothalamic-pituitary-adrenal (HPA) axis dysregulation, oxidative stress, and kynurenine pathway alterations might collectively disrupt neurotransmitter regulation and neuroplasticity, potentially manifesting as clinical symptoms. Specifically, cerebral inflammation in schizophrenia patients activates microglia to release pro-inflammatory factors, disrupting neuronal function and synaptic plasticity, which may manifest as clinical symptoms. Cytokine imbalance could influence schizophrenia pathology through its effects on dopamine and glutamate systems. Furthermore, the authors highlighted bidirectional interactions between neuroinflammation and chronic stress: sustained stress activates the HPA axis, increases cortisol release, and subsequently induces systemic inflammatory responses. Notably, current evidence remains inconclusive regarding whether the development of cognitive impairment (CI) in schizophrenia patients involves peripheral inflammation or neuroinflammatory alterations. In recent years, there has been a growing body of research exploring the relationship between cognitive function and inflammatory responses in patients with mental disorders. Increasing evidence suggests that inflammatory responses are closely related to cognitive impairment (CI) and may be an important mechanism underlying the occurrence of CI (10). Studies have found that the level of cognitive function in patients with bipolar disorder and depression disorder is related to inflammatory cytokines (11, 12). It has also been suggested that inflammation may be an important neuropathologic mechanism for cognitive decline and dementia in elderly patients (13). Studies on schizophrenia have also discovered a negative correlation between CRP (C-reactive protein, CRP) levels and cognitive function in individuals with schizophrenia (14, 15). In addition, when antipsychotic medications are combined with immunomodulators or anti-inflammatory treatments, cognitive improvement is more noticeable in people with schizophrenia (16). A review has concluded that CI in schizophrenia may be related to peripheral inflammation (17), when the body experiences inflammation, the central nervous system’s local inflammatory response overactivated the brain’s microglia, causing them to release high levels of inflammatory mediators like IL-6, TNF-α, IL-1β, and other cytokines, which impairs cognitive function (18–20). Therefore, the activation of inflammatory response and the dysregulation of the immune system may play an important role in schizophrenia CI, and peripheral inflammatory markers may be a simple and effective biological target for improving cognitive function in schizophrenia patients. However, Current research remains limited regarding the association between cognitive impairment and immune-inflammatory responses in schizophrenia, and traditional inflammatory cytokines often require complex techniques like ELISA require specialized equipment and trained personnel, posing significant technical and financial challenges. There is an urgent need to identify accessible and cost-effective inflammatory biomarkers for clinical studies. In recent years, Neutrophils and lymphocytes ratio (NLR), platelet and lymphocyte ratio (PLR), systemic immune inflammation index (systemic immune - inflammation index, SII index) [calculating formula for neutrophils (platelets lymphocyte ratio] such as a new type of periarteritis). These indicators are easy to evaluate, low cost, can be obtained from retrospective data, and can accurately represent the body’s inflammation, so they have been applied in more and more studies (21–23). Compared to traditional inflammation markers (e.g., IL-6, TNF-α, IL-1β, etc.), NLR, PLR, and SII are easier and cheaper to measure. These markers use simple blood test results (already done in most clinics) and don’t require extra costs or equipment. They are especially useful for large studies or areas with limited resources. Traditional markers like CRP mainly show short-term inflammation and can vary easily with external factors. In contrast, NLR, PLR, and SII track changes in common blood cells (neutrophils, lymphocytes, platelets) over time. These combined measures are more stable and give a fuller picture of immune system activity in mental disorders compared to single markers like CRP. Therefore, NLR, PLR and SII, as easily accessible, low-cost, more comprehensive and stable biomarkers, can be more easily integrated into routine clinical practice for early detection and monitoring of cognitive impairment in schizophrenic patients. This research investigates the relationship between NLR, PLR, SII index, and the risk of CI in schizophrenia patients through cross-sectional studies. Furthermore, it investigates the function of immunological inflammatory response in the etiology of CI in schizophrenia patients, providing theoretical evidence for future research. It provides a new idea and method for cognitive therapy of schizophrenic patients.

## 2 Data and methods

### 2.1 Research object

The subjects of the study were schizophrenia patients hospitalized in Minhang Mental Health Center from March 1, 2024, to October 31, 2024. The patients were enrolled according to admission criteria and divided into CI and CN (Cognitive normal) groups according to cognitive function level. The patients in the CI group were categorized into three groups: mild CI group, moderate CI group, and severe CI group.

### 2.2 Scheduling standards

Entry criteria: (1) Age 18-75 years old, regardless of gender; (2) It meets the diagnostic criteria of schizophrenia in ICD-10(International Classification of Disease-10, ICD-10); (3) Have sufficient audiovisual and understanding ability, no language communication barriers, and can complete the survey and cognitive function test (4); Obtaining informed consent.

Exclusion criteria: (1) patients with dementia, epilepsy, stroke, brain trauma, and other diseases that seriously affect cognitive function; (2) Long-term chronic infectious diseases, tumors, and other serious physical diseases, recent trauma, surgery, there are obvious infections; (3) Patients who have received immunosuppressants, non-steroidal anti-inflammatory drugs, antibiotics, glucocorticoids or MECT within the last 1 month; (4) Intellectual Disability, Down’s syndrome and dementia; (5) Women during pregnancy or breastfeeding; Tobacco, alcohol and other psychoactive substance abusers.

### 2.3 Ethical approval

The study followed the principles of the Declaration of Helsinki, and is approved by the Ethics Committee of Shanghai Minhang Mental Health Center (LW202401).

### 2.4 Clinical data collection

#### 2.4.1 General information questionnaire

The research group created the questionnaire, which included name, age, gender, marital status, education level, occupational status, length of hospital stay, prior history, family history, age of onset, course of disease, medication, etc. To analyze antipsychotic medication effects on inflammatory markers in schizophrenia, this study stratifies participants into three pharmacotherapy groups: Typical antipsychotic monotherapy (e.g., haloperidol), Atypical antipsychotic monotherapy (e.g., risperidone), Combination therapy (typical + atypical agents). Medication data were extracted from electronic medical records. All antipsychotic doses were standardized to chlorpromazine equivalents (CPZE) using validated conversion methods.

#### 2.4.2 Cognitive function assessment

Trained psychiatrists used a brief mental state examination (MMSE) to assess cognitive function. MMSE is one of the most prevalent and widely used CI assessment tools in the world, and is particularly suitable for rapid assessment of hospitalized patients. The scale contains five dimensions: orientation, memory, attention, numeracy, and language, and recall. MMSE has been shown to have good reliability and validity in patients with schizophrenia, and its advantages such as simple operation and easy access to coordination have been widely used in the assessment of cognitive function in psychiatric patients. With an overall MMSE score of 30, the cut-off point for screening for cognitive impairment in patients with schizophrenia is 24, and patients with an MMSE score of 24 and below are considered to have cognitive impairment (CI) (24).

#### 2.4.3 Laboratory index determination

Blood samples (5 mL) were collected from fasting patients via antecubital venipuncture under standardized phlebotomy protocols between 06:00–07:00 AM using EDTA-K2 vacuum tubes. Samples were mixed gently for 5 minutes, briefly remixed, and transported to the Clinical Laboratory of Shanghai Minhang Mental Health Center within 2 hours for complete blood count (CBC) analysis on the XN-1000 SA-01 automated hematology analyzer. Record the neutrophil count, lymphocyte count, monocyte count, and platelet count, then calculate the values of NLR, PLR, and SII index by the formula. All patients were required to fast for more than 12 hours before blood was extracted, high-protein, greasy food, and alcohol were prohibited the day before blood was extracted.

### 2.5 Statistical analysis

Statistical analyses were conducted using SPSS 26.0. Continuous variables were first assessed for normality via the Shapiro-Wilk test. As all continuous variables exhibited non-normal distributions (skewed), data were reported as median (interquartile range) [*M (P25, P75)*]. Categorical data were compared via chi-square (χ²) tests, presented as frequencies (%). Between-group differences in continuous variables used nonparametric tests (Mann-Whitney U/Kruskal-Wallis), with Bonferroni corrected pairwise comparisons (*P*<0.0167 for 3+ groups). Spearman correlation analyzed variable relationships. Statistical significance was defined as *P*<0.05.

## 3 Results

This study included 331 patients in total, 186 (56.2%) in the CN group and 145 (43.8%) in the CI group. Since all continuous variables are skewed, the median (interquartile range) *[M (P25, P75)]* is used to express them.

### 3.1 Comparison of general data between the two groups

There was no significant difference between the CN and CI groups in terms of gender, history of alcohol and tobacco use prior to hospitalization, family history of mental illness, history of diabetes or hypertension, BMI, first -generation or second-generation use of antipsychotic drugs, use of combination drugs, and uric acid. The two groups differed significantly in terms of age, marriage, education, age of first onset, and equivalent dose of chlorpromazine (*P* < 0.05). Compared with the CN group, schizophrenia patients with CI had a higher age, a lower proportion of high school or above education, a higher proportion of divorced and widowed patients, and a lower equivalent dose of chlorpromazine (**Table 1**).

**Table 1:** Comparison of general data between the CN and CI groups.

Terms	The cognitively normal	Cognitive Impairment	χ2/Z	*P*
Number (%)	186 (56.2)	145 (43.8)		
Male (n,%)	105 (56.5)	86 (59.3)	0.601	0.273
Age (years)	56 (47.8, 66.0)	62 (51.0, 69.0)	-2.626	0.009
High school and above (n,%)	94 (50.5)	52 (35.9)	7.118	0.008
Marriage (n,%)			14.987	0.002
unmarried	120 (64.5)	72 (49.7)		
married	34 (18.3)	21 (14.5)		
divorcee	27 (14.5)	44 (30.3)		
bereaved of one’s spouse (literary)	5 (2.7)	8 (5.5)		
History of smoking (n,%)	26 (14.0)	24 (16.6)	0.421	0.517
History of alcohol consumption (n,%)	10 (5.4)	12 (8.3)	1.104	0.293
Family history (n,%)	31 (16.7)	19 (13.2)	0.761	0.383
History of hypertension/diabetes (n,%)	166 (89.2)	117 (80.3)	4.813	0.103
Age at first incidence (years)	25 (19.8, 32.0)	28 (20.5, 39.0)	-2.589	0.01
Total duration of illness (years)	27 (21.0, 37.3)	28 (18.5, 38.5)	-0.01	0.992
BMI (Kg/m²)	24.4 (21.9, 27.3)	24.1 (21.6, 26.5)	-1.334	0.182
First-generation antipsychotics (n,%)	60 (32.3)	43 (29.7)	0.258	0.612
Second-generation antipsychotic (n,%)	165 (88.7)	126 (86.9)	0.252	0.616
Co-medication (n,%)	40 (21.5)	24 (16.6)	1.282	0.258
Chlorpromazine equivalent dose (mg/d) (d)	300 (200-400)	250 (200-400)	-2.497	0.013
Uric acid (umol/L)	339.7 (273.2-407.2)	321.3 (273.4-380.9)	-0.825	0.409

### 3.2 Comparison of laboratory indicators between the two groups

**Figure 1:**
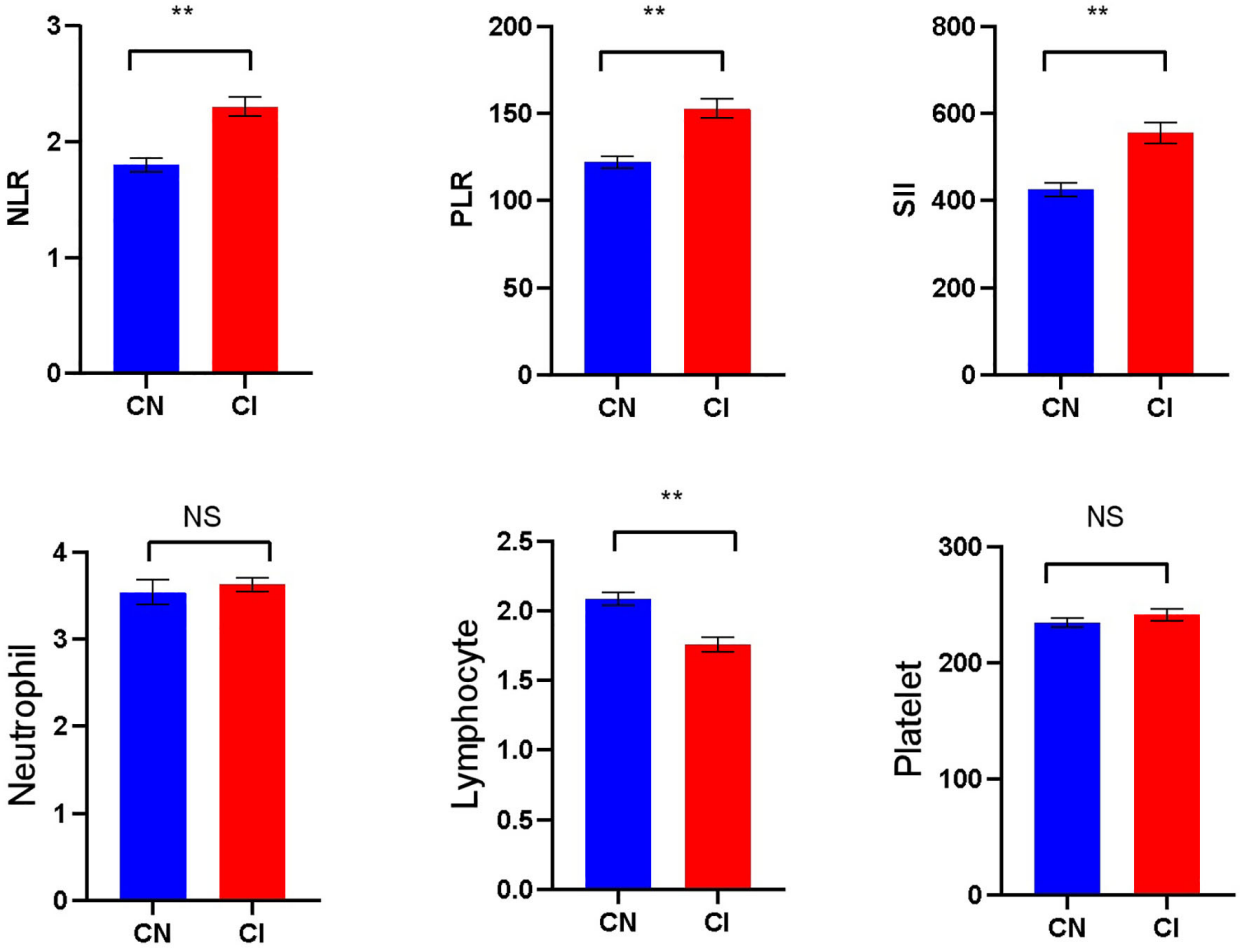
Comparison of laboratory indicators betweenthe CN and CI groups (** *p*<0.001; NS, No significant).

Significant differences were seen in NLR, PLR, SII, and lymphocytes levels between the CN and CI groups (*P* < 0.001). The NLR [2.00(1.62-2.70)], PLR [140(107.41-180.00)], and SII [498.75(363.61-640.79)] in the CI group were higher than the NLR [1.67 (1.24-2.22)], PLR 114.50 (91.13-147.56)], SII[397.73 (247.85-566.31)] in the CN group, whereas the lymphocytes 1.60 (1.30-2.15] in the CI group was lower than [the lymphocytes [2.10(1.70-2.50)] in the CN group. There was no significant difference in neutrophils and platelets counts between the two groups(*P*>0.05) (**Figure 1**).

### 3.3 Comparison of laboratory indicators in different degrees of CI group

**Figure 2:**
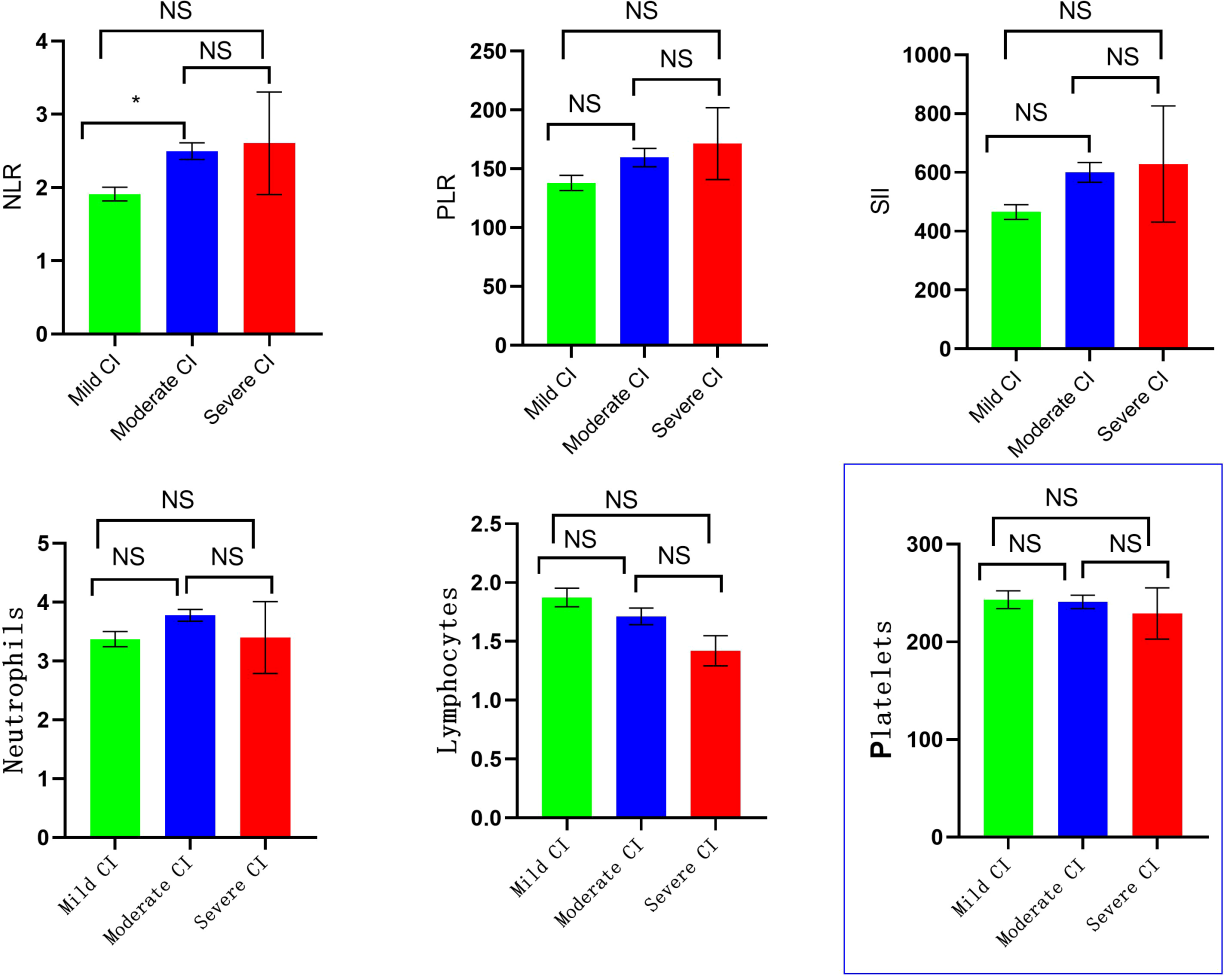
Comparison of laboratory indicators in different degrees of CI group. (* *p* < 0.0167, NS, No Significant).

There were statistically significant differences in the levels of NLR and SII among schizophrenia patients in the mild, moderate, and severe CI groups (*P*<0.05), but no significant differences in the levels of PLR, neutrophils, lymphocytes, and platelets(*P*>0.05). Bonferroni correction was applied to three pairwise comparisons (Mann-Whitney U) across three groups, with a corrected significance threshold of α = 0.05/3 = 0.0167. Group differences were considered statistically significant at *P* < 0.0167. It was found that there was a significant difference in NLR (P=0.002) only between patients in the mild CI and moderate CI groups, and the NLR [2.23 (1.78-2.93)] in the moderate CI group was higher than the NLR [1.77 (1.46-2.27)] in the mild CI group. There was no statistically significant differences between the remaining groups (*P > 0.0167*) (**Figure 2**).

### 3.4 Spearman correlation analysis of serum NLR, PLR, SII, and other indicators with cognitive function score

**Figure 3:**
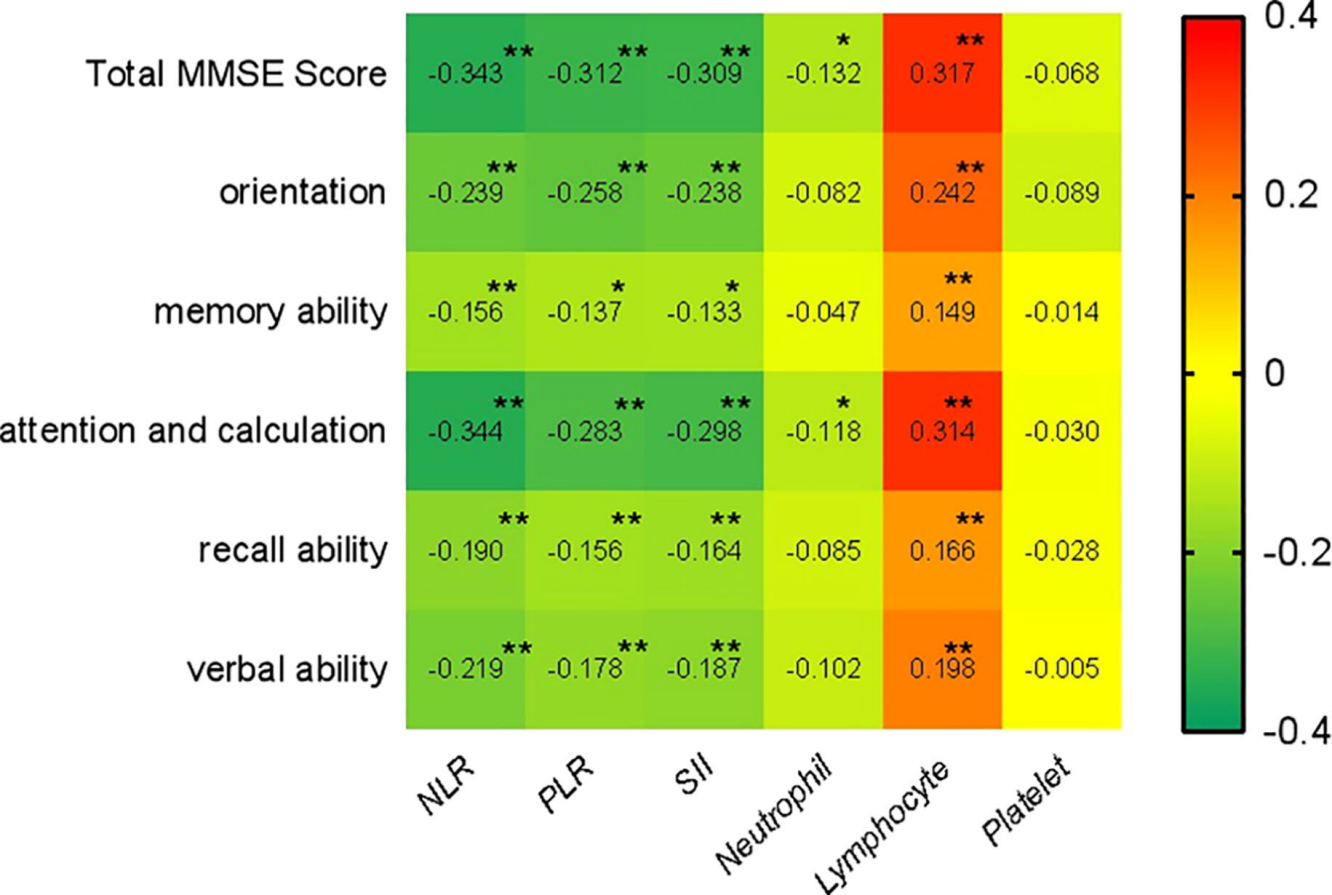
Spearman Spearman correlation analysis of NLR, PLR, and SII with cognitive function. (** *p* < 0.001, * *p* < 0.05).

The correlation between each research factor and cognitive function score was analyzed by Spearman correlation analysis. The findings demonstrated that lymphocytes had a significantly positive correlation with the overall score of cognitive function and scores of all dimensions, while NLR, PLR, and SII had a significantly negative correlation with these scores. Neutrophils were only negatively correlated with the total score of cognitive function, attention and computation scores. There was no significant correlation between platelets and the total score of cognitive function, as well as the scores of the various dimensions (**Figure 3**).

### 3.5 Correlation between serum NLR, PLR, SII, and other indicators and CI risk in schizophrenia

The correlation between different inflammatory markers and the CI risk were analyzed by the logistic regression method. With cognitive impairment (CI) status (0 = no CI, 1 = CI) as the dependent variable, and NLR, PLR, SII, neutrophils, lymphocytes, and platelets were each tested as independent variables in separate logistic regression models, the analysis results showed that without adjusting any covariates, the higher NLR(*OR*: 1.894, 95%*CI*: 1.435-2.499), PLR (*OR*: 1.011,95%CI: 1.006-1.016), SII (*OR*: 1.002, 95%*CI*: 1.001-1.003) were significantly associated with a higher CI risk, and higher lymphocytes (*OR*: 0.413, 95%*CI*: 0.281-0.605) were associated with a lower CI risk. After accounting for confounding factors such as sex, age, education, marriage, alcohol use, smoking history, age of first onset, total duration of schizophrenia, family history of psychosis, hypertension, diabetes, antipsychotic drug use (Typical antipsychotic monotherapy, Atypical antipsychotic monotherapy or Combination therapy), chlorpromazine equivalent dose (CPZE), BMI, uric acid, the results still showed a higher NLR(*OR*:2.033, 95%*CI*: 1.507-2.743), PLR (OR: 1.012, 95%CI: 1.007-1.018), SII (*OR*: 1.003, 95%*CI*:1.001-1.004) were significantly associated with a higher CI risk, and higher lymphocytes(*OR*: 0.367, 95%*CI*: 0.235-0.574) were associated with a lower CI risk. There was no significant correlation between neutrophils and platelets and the CI risk (**Table 2**). Stratified analyses of key confounders (age, illness duration, chlorpromazine equivalents, medication regimens [first generation antipsychotics, second-generation antipsychotics, or combination]) demonstrated no significant interactions with NLR, PLR, or SII (P value for interaction>0.05).The inflammatory marker-cognition associations maintained directional consistency across strata, confirming robustness (**Supplementary Tables 1**–**6**).

**Table 2:** Correlation between serum NLR, PLR, SII, and CI risk in schizophrenia.

Terms	*P*	*OR*	*95% CI*
Model 1			
NLR	<0.001	1.894	1.435-2.499
PLR	<0.001	1.011	1.006-1.016
SII	<0.001	1.002	1.001-1.003
Neutrophils	0.606	1.038	0.901-1.197
Lymphocytes	<0.001	0.413	0.281-0.605
Platelets	0.287	1.002	0.998-1.006
Model 2			
NLR	<0.001	2.033	1.507-2.743
PLR	<0.001	1.012	1.007-1.018
SII	<0.001	1.003	1.001-1.004
Neutrophils	0.453	1.059	0.912-1.229
Lymphocytes	<0.001	0.367	0.235-0.574
Platelets	0.113	1.003	0.999-1.008

### 3.6 Multiple linear regression analysis of NLR, PLR, SII, and other indicators and cognitive function score

Linear regression analyses were performed with MMSE score as the dependent variable, modeling NLR, PLR, SII, neutrophils, lymphocytes, and platelets each as the independent variable in separate models, the analysis showed that NLR, PLR, and SII were negatively correlated with the total cognitive function score and the scores of each dimension (*β* < 0, *p* < 0.001), and lymphocyte was positively correlated with the total cognitive function score and the scores of each dimension (*β* > 0, *p* < 0.001), when covariates were not adjusted. Neutrophils, platelets, and total cognitive function score and each dimension score were not significantly correlated. After accounting for confounding factors such as sex, age, education, marriage, alcohol use, smoking history, age of first onset, total duration of schizophrenia, family history of psychosis, hypertension, diabetes, antipsychotic drug use (Typical antipsychotic monotherapy, Atypical antipsychotic monotherapy or Combination therapy), chlorpromazine equivalent dose (CPZE), BMI, uric acid, the results still showed that NLR, PLR, and SII were negatively correlated with the total cognitive function score and each dimension (*β* < 0, *p* < 0.001), and lymphocytes were positively correlated with the total cognitive function score and each dimension (*β* > 0, *p* < 0.001). There was no significant correlation between neutrophils, platelets and total cognitive function score and each dimension score (**Table 3**). Variance inflation factor (VIF) analysis revealed values of 1.084 (NLR), 1.092 (PLR), 1.063 (SII), 1.019 (neutrophils), 1.163 (lymphocytes), and 1.068 (platelets), all below the threshold of 5, indicating no significant multicollinearity.

**Table 3:** Multiple linear regression analysis of NLR, PLR, SII and cognitive function score.

Terms	The total MMSE score		The orientation		Memory ability		Attention and calculatin		The recall ability		The verbal ability	
	*β*	*p*	*β*	*p*	*β*	*p*	*β*	*p*	*β*	*P*	*β*	*p*
Model 1												
NLR	-0.353	<0.001	-0.267	<0.001	-0.100	0.070	-0.352	<0.001	-0.185	0.001	-0.226	<0.001
PLR	-0.334	<0.001	-0.312	<0.001	-0.113	0.040	-0.301	<0.001	-0.178	0.001	-0.191	<0.001
SII	-0.319	<0.001	-0.252	<0.001	-0.110	0.046	-0.291	<0.001	-0.163	0.003	-0.232	<0.001
neutrophil	-0.058	0.294	-0.069	0.212	-0.004	0.936	-0.047	0.394	-0.014	0.805	-0.044	0.422
lymphocyte	0.306	<0.001	0.241	<0.001	0.134	0.015	0.292	<0.001	0.147	0.007	0.197	<0.001
Platelets	-0.043	0.436	-0.043	0.440	-0.041	0.459	-0.034	0.536	-0.002	0.977	-0.033	0.549
Model 2												
NLR	-0.353	<0.001	-0.271	<0.001	-0.113	0.047	-0.356	<0.001	-0.166	0.003	-0.238	<0.001
PLR	-0.344	<0.001	-0.327	<0.001	-0.126	0.027	-0.307	<0.001	-0.171	0.003	-0.209	<0.001
SII	-0.324	<0.001	-0.262	<0.001	-0.130	0.020	-0.290	<0.001	-0.156	0.005	-0.233	<0.001
neutrophil	-0.062	0.257	-0.068	0.214	-0.017	0.755	-0.044	0.434	-0.018	0.750	-0.040	0.467
lymphocyte	0.323	<0.001	0.259	<0.001	0.143	0.015	0.305	<0.001	0.122	0.038	0.236	<0.001
Platelets	-0.063	0.264	-0.061	0.274	-0.070	0.213	-0.049	0.389	-0.030	0.592	-0.027	0.631

### 3.7 ROC analysis for cognitive impairment prediction

ROC curves evaluated NLR, PLR, SII, neutrophils, lymphocytes, and platelets as predictors of cognitive impairment in schizophrenia. Significant predictors (*P*<0.05) included NLR (AUC=0.66, 95%CI 0.60–0.72), PLR (AUC=0.65, 95%CI 0.59–0.71), SII (AUC=0.65, 95%CI 0.59–0.71), and lymphocytes (AUC=0.34, 95%CI 0.28–0.40). NLR, PLR, and SII showed moderate diagnostic efficacy (AUC>0.65), with optimal cutoffs at 1.73 (NLR), 131.5 (PLR), and 403 (SII). Lymphocytes demonstrated inverse prediction (AUC<0.5). Neutrophils (AUC=0.55, *P*=0.11) and platelets (AUC=0.53, *P*=0.40) showed no predictive value (**Table 4**).

**Table 4:** ROC analysis of NLR, PLR, and SII for predicting cognitive impairment in schizophrenia.

Terms	AUC	Youden’s Index	Cutoff	Sensitivity	Specificity	95%*CI*		*P*
						Lower Limit	Upper Limit	
NLR	0.659	0.262	1.730	0.703	0.559	0.601	0.717	<0.001
PLR	0.652	0.260	131.5	0.593	0.667	0.593	0.711	<0.001
SII	0.650	0.256	403	0.724	0.532	0.592	0.709	<0.001
Neutrophils	0.551	3.15	3.15	0.676	0.435	0.489	0.613	0.111
Lymphocytes	0.339	3.65	3.65	0.014	0.995	0.279	0.399	<0.001
Platelets	0.527	236.5	236.5	0.531	0.548	0.464	0.590	0.403

## 4 Discussion

NLR, PLR, and SII are blood biomarkers reflecting the inflammatory state of the body, which have attracted much attention in recent years, and they are new research hotspots in the field of cognitive disorders. Compared to conventional cytokine biomarkers (IL-6, IL-1β, TNF α), hematological indices (NLR, PLR, SII) provide cost-effective clinical utility through routine complete blood count (CBC) testing, less subject to transient fluctuations, and stably capture multidimensional features of chronic immune dysregulation, enabling direct integration into standardized diagnostic protocols for inflammatory monitoring. NLR, PLR, and SII are cheaper and easier to obtain than traditional inflammatory factors including interleukin-6 (IL-6), interleukin-1β (IL-1β), and tumor necrosis factor-α (TNF-α). Additionally, neutrophils, lymphocytes, and platelets play a crucial role in the inflammatory response. When inflammation occurs in the body, neutrophils increase and activate the adaptive immune response mediated by lymphocytes, resulting in lymphocyte apoptosis, and then increasing the levels of NLR. NLR thus represents a balance between two complementary immune pathways (intrinsic and adaptive immunity) (25), is less affected by various other states, and is more stable than counting white blood cell subsets alone (26), with more reliable and accurate results. Platelets are non-specific inflammatory indicators that are commonly associated with hemostasis and thrombosis. Recent research has revealed that platelets play an essential role in the body’s inflammatory response and immunological regulation (27, 28). Neutrophils, platelets, and lymphocytes participate in different inflammatory or immune pathways in the body, while NLR, PLR, and SII combine these three complementary cells, so they are more reliable and predictive than evaluating the effects of neutrophils, platelets, or lymphocytes alone, because they can objectively reflect the balance of systemic inflammation and immunological response, are less impacted by confounding factors, and can be used to evaluate chronic systemic inflammation (29). In recent years, NLR, PLR, and SII have been gradually applied to the study of various psychiatric disorders (30–32). Some scholars have used inflammatory markers such as NLR and PLR in studies on schizophrenia patients, and have obtained meaningful findings. Multiple studies have found that peripheral blood lymphocyte counts are reduced and neutrophil counts and NLR are increased (33–36) in schizophrenia patients compared to controls, and higher NLR may be a risk factor for schizophrenia patients (37). In addition, studies have found that NLR levels are associated with positive symptoms of schizophrenia, but not with negative symptoms (38). Studies have also shown that NLR levels is higher in the acute phase of schizophrenia than in the remission phase, and there may be some differences in NLR in different disease stages (39, 40). Additionally, Chinese researchers have discovered that the NLR and PLR of schizophrenia patients are higher than those of the healthy control group, as well as the NLR of schizophrenia patients and the healthy control group is different significantly (41). All of the researchers mentioned above have found that schizophrenia patients have abnormal markers such as NLR and PLR, which may be linked to various symptoms and the development of schizophrenia. However, few studies have investigated whether cognitive impairment in schizophrenia patients is linked to neutrophils, lymphocytes, NLR, PLR, SII, and so on.

Previous research has shown that higher NLR is associated with an increased risk of mild cognitive impairment in AD (42). The AKL study also discovered that PD patients’ NLR was considerably higher than that of healthy people (43). In research on elderly patients with ischemic stroke, NLR levels were discovered to be considerably greater in the cognitively impaired group than in the healthy control group, and NLR levels were associated with the severity of cognitive impairment (44). Some domestic scholars have evaluated the relationship between NLR level and cognitive function of elderly people in Chinese communities and found that the NLR level of elderly people with mild cognitive impairment is significantly increased (45, 46). There have been few investigations on the relationship between PLR, SII levels and cognitive function. Study on diabetes have found that PLR levels are significantly associated with cognitive decline in patients with type 2 diabetes (47). Another study monitored SII and cognitive function of patients before and after orthopedic surgery found that patients with higher SII before surgery were more likely to have lower cognitive function after surgery (48). These studies have shown that NLR, PLR, and SII are associated with impaired cognitive function in a variety of disorders and may have an important role in the development of cognitive impairment. This is consistent with our findings that in the present study, we found that schizophrenia patients in the group with cognitive impairment had higher NLR, PLR, and SII than patients in the cognitively normal group, and correlation analyses and logistic regression modeling also found that elevated NLR, PLR, and SII were associated with higher cognitive impairment. After incorporating confounding factors such as age, total duration of illness, and medication use, the above results were still presented, suggesting that cognitive dysfunction in schizophrenic patients may be somehow associated with alterations in NLR, PLR, and SII. NLR, PLR, and SII, as a composite of neutrophil, lymphocyte, and platelet counts, may reflect peripheral inflammation in the organism to some extent. In recent years, more and more evidence has shown that peripheral inflammation can also cross the blood-brain barrier to induce central inflammation and activate microglia, thus leading to the occurrence of cognitive impairment (49). The cerebral cortex and hippocampus contain high levels of pro-inflammatory cytokines (PICs) and their receptors, so the hippocampus is more vulnerable to inflammatory attack, which will affect the occurrence and transmission of nerves, destroy synaptic plasticity, and lead to atrophy of dendritic branches. Resulting in impaired cognitive function and brain atrophy (50). The brain tissue of schizophrenia patients is in a state of immunological inflammation, resulting in the release of a range of cytokines, which not only regulate cell growth and differentiation, but also participate in the body’s inflammatory damage process, while activating microglia of the central system, enhancing the synthesis of neurotoxic mediators (inflammatory factors and free radicals, etc.), and activating neurons’ inflammatory response. It leads to reduced expression of neuroplasticity protein, which is closely related to the pathogenesis of cognitive impairment (51–54). A growing body of research suggests that cognitive function is mediated by bidirectional interactions between the brain and the immune system (9, 55). Stress-induced activation of the HPA axis releases glucocorticoids, which modulate immune cell activity and inflammatory factor secretion. Reverse causality—cognitive impairment driving immune dysregulation—may also occur: neuroimmunoregulatory dysfunction in cognitively impaired patients may aberrantly activate peripheral immunity, elevating the neutrophil-to-lymphocyte ratio (NLR), platelet-to lymphocyte ratio (PLR), and systemic immune-inflammation index (SII). As across-sectional design, this study cannot establish temporal precedence between inflammation and cognitive decline. Longitudinal studies tracking marker-trajectory and cognitive changes are needed to disentangle causality. In addition, Previous studies have suggested that NLR, PLR, and SII may be associated with systemic oxidative injury through the following mechanisms: (i) neutrophil activation accompanied by ROS Release (56); (ii) platelet-derived ROS promote oxidative damage to the vascular endothelium (57); (iii) oxidative stress induced lymphocyte apoptosis may amplify NLR/PLR abnormalities (58). Oxidative stress markers were not directly measured in this study, It is difficult to fully distinguish whether these marker changes stem primarily from peripheral inflammation, systemic oxidative stress, or their combination. Future studies should employ multi-omics approaches to clarify pathways.

In this study, multiple dimensions of cognitive function were correlated, and it was found that NLR, PLR, and SII were negatively correlated with the patients’ verbal ability, memory ability, attention and calculation ability, and orientation scores. This suggests that NLR, PLR, and SII may be associated with impaired cognitive function across multiple dimensions. This is consistent with the study of Marsland et al. (59) who discovered that peripheral inflammation is related to a reduction in white matter, cerebral cortex, cortical surface area, and hippocampal volume in as well as declines in language, short-term memory, executive function, and spatial reasoning ability. Interestingly, our study found lymphocytes positively correlated with multidomain cognition, suggesting a protective role in schizophrenia. Lymphocytes—key immune components including helper T (Th) and regulatory T (Treg) cells—mediate dual functions: pro-inflammatory Th17 cells drive neuroinflammation, while Tregs suppress excessive immunity, protect neurons, and preserve cognition (60, 61). We hypothesize: In stable immunity (e.g., chronic phase), Tregs dominate, dampening inflammation and safeguarding cognitive domains. In immune imbalance (e.g., acute exacerbation), Th17 overactivation disrupts this balance, inducing cognitive decline. Notably, this protective potential reflects lymphocyte subtype dynamics—a nuance obscured in our study by measuring total lymphocytes (no Treg/Th17 distinction). Future studies should dissect subtype-specific roles to clarify protective mechanisms. Our study also found that neutrophils were only associated with total cognitive function scores, attention and numeracy, while platelets were not significantly associated with any of the cognitive dimensions. However, previous studies have suggested that platelets may also have an important role in the development of cognitive impairment. One study found that intracerebral white matter lesions and cognitive decline in patients with vascular dementia were associated with platelet activation (62). We speculate there are the following reasons: First, platelet activation may not necessarily exhibit quantitative changes. Second, PLR, NLR, SII—as composite indices—better reflect inflammatory-immune dysregulation by balancing pro-inflammatory (platelets/neutrophils) and anti-inflammatory (lymphocytes) components, minimizing confounding bias. Furthermore, differences in study population, sample size, and cross-sectional design (short observation time) may introduce data inaccuracies. Future studies should integrate longitudinal platelet function assays (e.g., mitochondrial ROS), expand sample size and average multiple measurements to reduce bias, and clarify causality.

In this study, NLR, PLR, SII were compared across CI severity: only NLR differed between mild vs moderate CI (Bonferroni-corrected p<0.0167), with no PLR, SII group differences. We hypothesized the following reasons: 1. Dynamic inflammation: inflammation peaks during the normal → mild CI transition (e.g., microglia hyperactivation, blood-brain barrier disruption), which results in a significant difference between normal and CI; in the moderate/severe phase, non-inflammatory mechanisms (neuronal loss, synaptic dysfunction) prevail due to neuroprotection/immunosuppression, thus blurring the mild-moderate-severe intergroup Differences. 2. Sample/statistical limitations: within groups, sample sizes may be small, reducing statistical power. 3. Lymphocyte subtype confounding: the total number of lymphocytes masks the opposing roles of Th17 (pro- inflammatory) and Treg (anti-inflammatory), which may affect the results. Future studies should increase each group sizes and longitudinally track NLR, PLR, SII alongside neuroinflammatory markers to dissect their stage specific trajectories in CI.

In this study, ROC analysis was conducted to predict cognitive impairment (CI) using NLR, PLR, SII. It was found that serum NLR/PLR/SII showed superior predictive efficacy for CI in schizophrenia, lymphocytes fair efficacy, and neutrophils/platelets poor efficacy. ROC curves yielded cutoffs of 1.730 (NLR), 131.5 (PLR), 403 SII), and 3.65 (lymphocytes). Regarding (the critical values for screening cognitive impairment, there are few relevant studies, and different studies report different critical values due to differences in sample characteristics, testing methods, etc. If NLR, PLR, and SII are validated as screening thresholds for cognitive impairment, If NLR, PLR and SII are recognized as screening thresholds for cognitive dysfunction, they will be valuable for:

1. Diagnostic aid: testing these markers at schizophrenia diagnosis can identify early-stage CI-risk patients (those with elevated NLR/PLR/SII should be prioritized for cognitive interventions).
2. Disease monitoring: regular testing assesses CI progression and guides treatment adjustment (persistently elevated markers may indicate worsening impairment requiring intensified interventions).
3. Treatment evaluation: post-treatment marker decreases may reflect cognitive improvement. Future research should conduct larger multicenter studies should validate screening thresholds in different populations, clinical situations and scenarios to optimize the use of markers in screening and early intervention for CI in schizophrenia.

This study has limitations: First, single-center/small-sample bias: All participants were recruited from one psychiatric hospital, limiting generalizability and necessitating future multicenter replication with expanded samples. Second, assessment bias: Cognitive scale administrations lacked rater blinding, potentially introducing observer bias despite standardized training. Third, cross-sectional design constraints: Baseline data preclude causal inference between inflammatory markers and CI—longitudinal studies tracking NLR, PLR, SII alongside repeated cognitive assessments are needed to establish temporal relationships. Fourth, mechanistic ambiguity: NLR, PLR, SII reflect both peripheral inflammation and systemic oxidative stress, which current methods cannot disentangle; future studies should employ multi-omics approaches to clarify pathways. Fifth, cognitive assessment limitations: While MMSE enabled rapid CI screening, it missed schizophrenia-specific deficits in executive function and social cognition—adopting the MCCB (MATRICS Consensus Cognitive Battery) in future studies will better capture multidomain impairments. However, this study offers preliminary evidence for future longitudinal investigations, wherein patients could be stratified by clinical characteristics to design targeted follow-ups.

## Data Availability

The raw data supporting the conclusions of this article will be made available by the authors, without undue reservation.
