# Supplementary material for: Neutrophil-to-lymphocyte ratio; platelet-to-lymphocyte ratio; systemic immune-inflammatory Index: inflammatory indicators of cognitive impairment in schizophrenia patients
Source: Front Psychiatry. 2025 Apr 11;16:1552451. doi: 10.3389/fpsyt.2025.1552451 (PMC12022758; doi:10.3389/fpsyt.2025.1552451)
Supplement: Supplementary file 1 [file DataSheet1.pdf]

**Table 1 Subgroup analysis (NLR)**

| Variables | n (%) | OR (95%CI) | P for interaction |
|---|---|---|---|
| All patients | 331 (100.00) | 1.89 (1.44 ~ 2.50) | |
| **Sex** | | | 0.066 |
| Male | 191 (57.70) | 1.57 (1.13 ~ 2.16) | |
| Female | 140 (42.30) | 2.69 (1.65 ~ 4.38) | |
| **Education** | | | 0.525 |
| Primary school | 44 (13.29) | 1.59 (0.73 ~ 3.46) | |
| Middle school | 141 (42.60) | 2.06 (1.33 ~ 3.18) | |
| High school | 111 (33.53) | 2.15 (1.28 ~ 3.61) | |
| University | 35 (10.57) | 1.23 (0.69 ~ 2.21) | |
| **Marital status** | | | 0.076 |
| Unmarried | 192 (58.01) | 1.95 (1.36 ~ 2.80) | |
| Married | 55 (16.62) | 1.11 (0.66 ~ 1.86) | |
| Divorced | 71 (21.45) | 3.60 (1.67 ~ 7.75) | |
| Widowed | 13 (3.93) | 1.35 (0.11 ~ 17.24) | |
| **Alcohol history** | | | 0.785 |
| Yes | 22 (6.65) | 2.21 (0.68 ~ 7.21) | |
| No | 309 (93.35) | 1.87 (1.41 ~ 2.50) | |
| **Smoking history** | | | 0.705 |
| Yes | 50 (15.11) | 2.15 (1.02 ~ 4.57) | |
| No | 281 (84.89) | 1.85 (1.37 ~ 2.49) | |
| **Medical history** | | | 0.433 |
| None | 48 (14.50) | 3.99 (1.12 ~ 14.16) | |
| Hypertension | 172 (51.96) | 1.59 (1.13 ~ 2.26) | |
| Diabetes | 75 (22.66) | 2.03 (1.14 ~ 3.62) | |
| Hypertension + Diabetes | 36 (10.88) | 2.34 (0.93 ~ 5.92) | |

| Variables | n (%) | OR (95%CI) | P for interaction |
|---|---|---|---|
| **Family history** | | | 0.394 |
| Yes | 50 (15.15) | 1.55 (0.93 ~ 2.59) | |
| No | 280 (84.85) | 2.03 (1.48 ~ 2.80) | |
| **Medication** | | | 0.883 |
| Typical antipsychotics | 40 (12.08) | 1.67 (0.87 ~ 3.20) | |
| Atypical antipsychotics | 229 (69.18) | 1.91 (1.35 ~ 2.71) | |
| Combination therapy | 62 (18.73) | 2.11 (1.11 ~ 4.00) | |
| **Chlorpromazine equivalent dose** | | | 0.961 |
| <300 mg/day | 156 (47.13) | 1.99 (1.31 ~ 3.03) | |
| 300–600 mg/day | 163 (49.24) | 1.86 (1.23 ~ 2.82) | |
| >600 mg/day | 12 (3.63) | 2.11 (0.74 ~ 6.04) | |
| **Age group** | | | 0.145 |
| 18–44 years | 48 (14.50) | 3.75 (1.28 ~ 10.97) | |
| 45–60 years | 125 (37.76) | 1.39 (0.92 ~ 2.10) | |
| 61–75 years | 158 (47.73) | 2.04 (1.34 ~ 3.11) | |
| **Disease duration** | | | 0.615 |
| <5 years | 9 (2.72) | 1.28 (0.05 ~ 32.28) | |
| 5–10 years | 15 (4.53) | 6.39 (0.74 ~ 55.03) | |
| 11–20 years | 53 (16.01) | 1.64 (0.80 ~ 3.36) | |
| >20 years | 254 (76.74) | 1.91 (1.40 ~ 2.61) | |
| **Serum uric acid** | | | 0.867 |
| Normal | 268 (80.97) | 1.92 (1.41 ~ 2.62) | |
| Hyperuricemia | 63 (19.03) | 1.81 (0.94 ~ 3.48) | |
| **BMI category** | | | 0.436 |
| Underweight | 14 (4.23) | 0.99 (0.26 ~ 3.82) | |

| Variables | n (%) | OR (95%CI) | P for interaction |
|---|---|---|---|
| Normal | 135 (40.79) | 1.69 (1.16 ~ 2.47) | |
| Overweight | 182 (54.98) | 2.22 (1.44 ~ 3.43) | |

OR: Odds Ratio, CI: Confidence Interval

**Table 2 Subgroup analysis (PLR)**

| Variables | n (%) | OR (95%CI) | P for interaction |
|---|---|---|---|
| All patients | 331 (100.00) | 1.01 (1.01 ~ 1.02) | |
| **Sex** | | | 0.700 |
| Male | 191 (57.70) | 1.01 (1.00 ~ 1.02) | |
| Female | 140 (42.30) | 1.01 (1.00 ~ 1.02) | |
| **Education** | | | 0.902 |
| Primary school | 44 (13.29) | 1.01 (1.00 ~ 1.02) | |
| Middle school | 141 (42.60) | 1.01 (1.00 ~ 1.02) | |
| High school | 111 (33.53) | 1.01 (1.00 ~ 1.02) | |
| University | 35 (10.57) | 1.01 (1.00 ~ 1.03) | |
| **Marital status** | | | 0.313 |
| Unmarried | 192 (58.01) | 1.01 (1.00 ~ 1.02) | |
| Married | 55 (16.62) | 1.01 (0.99 ~ 1.02) | |
| Divorced | 71 (21.45) | 1.02 (1.01 ~ 1.03) | |
| Widowed | 13 (3.93) | 1.03 (0.99 ~ 1.07) | |
| **Alcohol history** | | | 0.993 |
| Yes | 22 (6.65) | 1.01 (0.99 ~ 1.03) | |
| No | 309 (93.35) | 1.01 (1.01 ~ 1.02) | |
| **Smoking history** | | | 0.406 |
| Yes | 50 (15.11) | 1.02 (1.00 ~ 1.03) | |
| No | 281 (84.89) | 1.01 (1.01 ~ 1.02) | |

| Variables | n (%) | OR (95%CI) | P for interaction |
|---|---|---|---|
| **Medical history** | | | 0.371 |
| None | 48 (14.50) | 1.02 (1.00 ~ 1.04) | |
| Hypertension | 172 (51.96) | 1.01 (1.00 ~ 1.02) | |
| Diabetes | 75 (22.66) | 1.01 (1.00 ~ 1.02) | |
| Hypertension + Diabetes | 36 (10.88) | 1.02 (1.00 ~ 1.04) | |
| **Family history** | | | 0.626 |
| Yes | 50 (15.15) | 1.01 (1.00 ~ 1.03) | |
| No | 280 (84.85) | 1.01 (1.01 ~ 1.02) | |
| **Medication** | | | 0.715 |
| Typical antipsychotics | 40 (12.08) | 1.01 (1.00 ~ 1.02) | |
| Atypical antipsychotics | 229 (69.18) | 1.01 (1.01 ~ 1.02) | |
| Combination therapy | 62 (18.73) | 1.01 (1.00 ~ 1.02) | |
| **Chlorpromazine equivalent dose** | | | 0.632 |
| <300 mg/day | 156 (47.13) | 1.01 (1.00 ~ 1.02) | |
| 300–600 mg/day | 163 (49.24) | 1.01 (1.01 ~ 1.02) | |
| >600 mg/day | 12 (3.63) | 1.00 (0.99 ~ 1.02) | |
| **Age group** | | | 0.601 |
| 18–44 years | 48 (14.50) | 1.02 (1.00 ~ 1.04) | |
| 45–60 years | 125 (37.76) | 1.01 (1.00 ~ 1.02) | |
| 61–75 years | 158 (47.73) | 1.01 (1.00 ~ 1.02) | |
| **Disease duration** | | | 0.243 |
| <5 years | 9 (2.72) | 1.01 (0.97 ~ 1.06) | |
| 5–10 years | 15 (4.53) | 1.06 (0.99 ~ 1.13) | |
| 11–20 years | 53 (16.01) | 1.01 (1.00 ~ 1.02) | |
| >20 years | 254 (76.74) | 1.01 (1.01 ~ 1.02) | |

| Variables | n (%) | OR (95%CI) | P for interaction |
|---|---|---|---|
| **Serum uric acid** | | | 0.568 |
| Normal | 268 (80.97) | 1.01 (1.01 ~ 1.02) | |
| Hyperuricemia | 63 (19.03) | 1.01 (0.99 ~ 1.02) | |
| **BMI category** | | | 0.475 |
| Underweight | 14 (4.23) | 1.01 (0.99 ~ 1.03) | |
| Normal | 135 (40.79) | 1.01 (1.01 ~ 1.02) | |
| Overweight | 182 (54.98) | 1.01 (1.00 ~ 1.01) | |

OR: Odds Ratio, CI: Confidence Interval

**Table 3 Subgroup analysis (SII)**

| Variables | n (%) | OR (95%CI) | P for interaction |
|---|---|---|---|
| All patients | 331 (100.00) | 1.00 (1.00 ~ 1.00) | |
| **Sex** | | | 0.590 |
| Male | 191 (57.70) | 1.00 (1.00 ~ 1.00) | |
| Female | 140 (42.30) | 1.00 (1.00 ~ 1.00) | |
| **Education** | | | 0.712 |
| Primary school | 44 (13.29) | 1.00 (1.00 ~ 1.00) | |
| Middle school | 141 (42.60) | 1.00 (1.00 ~ 1.00) | |
| High school | 111 (33.53) | 1.00 (1.00 ~ 1.00) | |
| University | 35 (10.57) | 1.00 (1.00 ~ 1.01) | |
| **Marital status** | | | 0.498 |
| Unmarried | 192 (58.01) | 1.00 (1.00 ~ 1.00) | |
| Married | 55 (16.62) | 1.00 (1.00 ~ 1.00) | |
| Divorced | 71 (21.45) | 1.00 (1.00 ~ 1.01) | |
| Widowed | 13 (3.93) | 1.00 (0.99 ~ 1.01) | |
| **Alcohol history** | | | 0.686 |

| Variables | n (%) | OR (95%CI) | P for interaction |
|---|---|---|---|
| Yes | 22 (6.65) | 1.00 (1.00 ~ 1.01) | |
| No | 309 (93.35) | 1.00 (1.00 ~ 1.00) | |
| **Smoking history** | | | 0.892 |
| Yes | 50 (15.11) | 1.00 (1.00 ~ 1.00) | |
| No | 281 (84.89) | 1.00 (1.00 ~ 1.00) | |
| **Medical history** | | | 0.337 |
| None | 48 (14.50) | 1.00 (1.00 ~ 1.01) | |
| Hypertension | 172 (51.96) | 1.00 (1.00 ~ 1.00) | |
| Diabetes | 75 (22.66) | 1.00 (1.00 ~ 1.00) | |
| Hypertension + Diabetes | 36 (10.88) | 1.00 (1.00 ~ 1.01) | |
| **Family history** | | | 0.812 |
| Yes | 50 (15.15) | 1.00 (1.00 ~ 1.00) | |
| No | 280 (84.85) | 1.00 (1.00 ~ 1.00) | |
| **Medication** | | | 0.356 |
| Typical antipsychotics | 40 (12.08) | 1.00 (1.00 ~ 1.00) | |
| Atypical antipsychotics | 229 (69.18) | 1.00 (1.00 ~ 1.00) | |
| Combination therapy | 62 (18.73) | 1.00 (1.00 ~ 1.00) | |
| **Chlorpromazine equivalent dose** | | | 0.536 |
| <300 mg/day | 156 (47.13) | 1.00 (1.00 ~ 1.00) | |
| 300–600 mg/day | 163 (49.24) | 1.00 (1.00 ~ 1.00) | |
| >600 mg/day | 12 (3.63) | 1.00 (1.00 ~ 1.01) | |
| **Age group** | | | 0.159 |
| 18–44 years | 48 (14.50) | 1.01 (1.00 ~ 1.01) | |
| 45–60 years | 125 (37.76) | 1.00 (1.00 ~ 1.00) | |
| 61–75 years | 158 (47.73) | 1.00 (1.00 ~ 1.00) | |

| Variables | n (%) | OR (95%CI) | P for interaction |
|---|---|---|---|
| **Disease duration** | | | 0.246 |
| <5 years | 9 (2.72) | 1.00 (0.99 ~ 1.01) | |
| 5–10 years | 15 (4.53) | 1.01 (1.00 ~ 1.02) | |
| 11–20 years | 53 (16.01) | 1.00 (1.00 ~ 1.00) | |
| >20 years | 254 (76.74) | 1.00 (1.00 ~ 1.00) | |
| **Serum uric acid** | | | 0.385 |
| Normal | 268 (80.97) | 1.00 (1.00 ~ 1.00) | |
| Hyperuricemia | 63 (19.03) | 1.00 (1.00 ~ 1.00) | |
| **BMI category** | | | 0.674 |
| Underweight | 14 (4.23) | 1.00 (1.00 ~ 1.00) | |
| Normal | 135 (40.79) | 1.00 (1.00 ~ 1.00) | |
| Overweight | 182 (54.98) | 1.00 (1.00 ~ 1.00) | |

OR: Odds Ratio, CI: Confidence Interval

**Table 4 Subgroup analysis (Neutrophils)**

| Variables | n (%) | OR (95%CI) | P for interaction |
|---|---|---|---|
| All patients | 331 (100.00) | 1.04 (0.90 ~ 1.20) | |
| **Sex** | | | 0.614 |
| Male | 191 (57.70) | 1.10 (0.83 ~ 1.46) | |
| Female | 140 (42.30) | 1.02 (0.87 ~ 1.19) | |
| **Education** | | | 0.169 |
| Primary school | 44 (13.29) | 0.91 (0.54 ~ 1.55) | |
| Middle school | 141 (42.60) | 1.39 (0.99 ~ 1.97) | |
| High school | 111 (33.53) | 0.96 (0.78 ~ 1.18) | |
| University | 35 (10.57) | 1.46 (0.79 ~ 2.67) | |
| **Marital status** | | | 0.059 |

| Variables | n (%) | OR (95%CI) | P for interaction |
|---|---|---|---|
| Unmarried | 192 (58.01) | 1.04 (0.89 ~ 1.21) | |
| Married | 55 (16.62) | 1.03 (0.58 ~ 1.82) | |
| Divorced | 71 (21.45) | 1.64 (0.91 ~ 2.97) | |
| Widowed | 13 (3.93) | 0.15 (0.02 ~ 1.19) | |
| **Alcohol history** | | | 0.791 |
| Yes | 22 (6.65) | 0.95 (0.50 ~ 1.81) | |
| No | 309 (93.35) | 1.04 (0.90 ~ 1.20) | |
| **Smoking history** | | | 0.786 |
| Yes | 50 (15.11) | 0.97 (0.58 ~ 1.61) | |
| No | 281 (84.89) | 1.04 (0.90 ~ 1.21) | |
| **Medical history** | | | 0.211 |
| None | 48 (14.50) | 1.08 (0.56 ~ 2.09) | |
| Hypertension | 172 (51.96) | 0.96 (0.81 ~ 1.15) | |
| Diabetes | 75 (22.66) | 1.44 (0.91 ~ 2.28) | |
| Hypertension + Diabetes | 36 (10.88) | 1.74 (0.78 ~ 3.88) | |
| **Family history** | | | 0.236 |
| Yes | 50 (15.15) | 1.40 (0.83 ~ 2.35) | |
| No | 280 (84.85) | 1.02 (0.88 ~ 1.17) | |
| **Medication** | | | 0.458 |
| Typical antipsychotics | 40 (12.08) | 0.78 (0.40 ~ 1.51) | |
| Atypical antipsychotics | 229 (69.18) | 1.03 (0.89 ~ 1.20) | |
| Combination therapy | 62 (18.73) | 1.30 (0.80 ~ 2.12) | |
| **Chlorpromazine equivalent dose** | | | 0.193 |
| <300 mg/day | 156 (47.13) | 0.97 (0.82 ~ 1.14) | |
| 300–600 mg/day | 163 (49.24) | 1.23 (0.89 ~ 1.68) | |

| Variables | n (%) | OR (95%CI) | P for interaction |
|---|---|---|---|
| >600 mg/day | 12 (3.63) | 2.09 (0.61 ~ 7.15) | |
| **Age group** | | | 0.104 |
| 18–44 years | 48 (14.50) | 1.60 (0.97 ~ 2.63) | |
| 45–60 years | 125 (37.76) | 0.95 (0.76 ~ 1.17) | |
| 61–75 years | 158 (47.73) | 1.17 (0.85 ~ 1.63) | |
| **Disease duration** | | | 0.284 |
| <5 years | 9 (2.72) | 0.17 (0.01 ~ 2.48) | |
| 5–10 years | 15 (4.53) | 1.66 (0.55 ~ 4.98) | |
| 11–20 years | 53 (16.01) | 1.12 (0.65 ~ 1.96) | |
| >20 years | 254 (76.74) | 1.03 (0.89 ~ 1.19) | |
| **Serum uric acid** | | | 0.963 |
| Normal | 268 (80.97) | 1.04 (0.90 ~ 1.21) | |
| Hyperuricemia | 63 (19.03) | 1.03 (0.62 ~ 1.71) | |
| **BMI category** | | | 0.013 |
| Underweight | 14 (4.23) | 0.50 (0.12 ~ 2.00) | |
| Normal | 135 (40.79) | 1.28 (0.90 ~ 1.82) | |
| Overweight | 182 (54.98) | 1.33 (0.99 ~ 1.79) | |

OR: Odds Ratio, CI: Confidence Interval

**Table 5 Subgroup analysis (Lymphocytes)**

| Variables | n (%) | OR (95%CI) | P for interaction |
|---|---|---|---|
| All patients | 331 (100.00) | 0.41 (0.28 ~ 0.61) | |
| **Sex** | | | 0.738 |
| Male | 191 (57.70) | 0.44 (0.26 ~ 0.73) | |
| Female | 140 (42.30) | 0.38 (0.21 ~ 0.69) | |
| **Education** | | | 0.763 |

| Variables | n (%) | OR (95%CI) | P for interaction |
|---|---|---|---|
| Primary school | 44 (13.29) | 0.55 (0.20 ~ 1.54) | |
| Middle school | 141 (42.60) | 0.41 (0.23 ~ 0.73) | |
| High school | 111 (33.53) | 0.35 (0.17 ~ 0.73) | |
| University | 35 (10.57) | 0.68 (0.22 ~ 2.10) | |
| **Marital status** | | | 0.745 |
| Unmarried | 192 (58.01) | 0.45 (0.26 ~ 0.78) | |
| Married | 55 (16.62) | 0.51 (0.21 ~ 1.26) | |
| Divorced | 71 (21.45) | 0.32 (0.15 ~ 0.68) | |
| Widowed | 13 (3.93) | 0.20 (0.02 ~ 1.87) | |
| **Alcohol history** | | | 0.520 |
| Yes | 22 (6.65) | 0.25 (0.05 ~ 1.23) | |
| No | 309 (93.35) | 0.42 (0.29 ~ 0.63) | |
| **Smoking history** | | | 0.442 |
| Yes | 50 (15.11) | 0.29 (0.11 ~ 0.79) | |
| No | 281 (84.89) | 0.44 (0.29 ~ 0.66) | |
| **Medical history** | | | 0.835 |
| None | 48 (14.50) | 0.29 (0.09 ~ 0.96) | |
| Hypertension | 172 (51.96) | 0.39 (0.23 ~ 0.68) | |
| Diabetes | 75 (22.66) | 0.53 (0.25 ~ 1.12) | |
| Hypertension + Diabetes | 36 (10.88) | 0.48 (0.16 ~ 1.44) | |
| **Family history** | | | 0.337 |
| Yes | 50 (15.15) | 0.25 (0.08 ~ 0.79) | |
| No | 280 (84.85) | 0.45 (0.30 ~ 0.68) | |
| **Medication** | | | 0.648 |
| Typical antipsychotics | 40 (12.08) | 0.25 (0.08 ~ 0.82) | |

| Variables | n (%) | OR (95%CI) | P for interaction |
|---|---|---|---|
| Atypical antipsychotics | 229 (69.18) | 0.45 (0.28 ~ 0.71) | |
| Combination therapy | 62 (18.73) | 0.41 (0.17 ~ 1.01) | |
| **Chlorpromazine equivalent dose** | | | 0.969 |
| <300 mg/day | 156 (47.13) | 0.42 (0.24 ~ 0.71) | |
| 300–600 mg/day | 163 (49.24) | 0.38 (0.21 ~ 0.68) | |
| >600 mg/day | 12 (3.63) | 0.43 (0.06 ~ 2.98) | |
| **Age group** | | | 0.829 |
| 18–44 years | 48 (14.50) | 0.38 (0.12 ~ 1.15) | |
| 45–60 years | 125 (37.76) | 0.50 (0.27 ~ 0.94) | |
| 61–75 years | 158 (47.73) | 0.40 (0.22 ~ 0.71) | |
| **Disease duration** | | | 0.384 |
| <5 years | 9 (2.72) | 0.03 (0.00 ~ 7.74) | |
| 5–10 years | 15 (4.53) | 0.12 (0.01 ~ 1.28) | |
| 11–20 years | 53 (16.01) | 0.45 (0.19 ~ 1.09) | |
| >20 years | 254 (76.74) | 0.41 (0.26 ~ 0.65) | |
| **Serum uric acid** | | | 0.418 |
| Normal | 268 (80.97) | 0.38 (0.25 ~ 0.59) | |
| Hyperuricemia | 63 (19.03) | 0.56 (0.25 ~ 1.21) | |
| **BMI category** | | | 0.695 |
| Underweight | 14 (4.23) | 0.55 (0.10 ~ 2.85) | |
| Normal | 135 (40.79) | 0.35 (0.19 ~ 0.65) | |
| Overweight | 182 (54.98) | 0.48 (0.28 ~ 0.82) | |

OR: Odds Ratio, CI: Confidence Interval

**Table 6 Subgroup analysis (Platelets)**

| Variables | n (%) | OR (95%CI) | P for interaction |
|---|---|---|---|
| All patients | 331 (100.00) | 1.00 (1.00 ~ 1.01) | |
| **Sex** | | | 0.846 |
| Male | 191 (57.70) | 1.00 (1.00 ~ 1.01) | |
| Female | 140 (42.30) | 1.00 (1.00 ~ 1.01) | |
| **Education** | | | 0.356 |
| Primary school | 44 (13.29) | 0.99 (0.98 ~ 1.01) | |
| Middle school | 141 (42.60) | 1.00 (1.00 ~ 1.01) | |
| High school | 111 (33.53) | 1.00 (1.00 ~ 1.01) | |
| University | 35 (10.57) | 1.01 (1.00 ~ 1.03) | |
| **Marital status** | | | 0.884 |
| Unmarried | 192 (58.01) | 1.00 (1.00 ~ 1.01) | |
| Married | 55 (16.62) | 1.00 (0.99 ~ 1.01) | |
| Divorced | 71 (21.45) | 1.00 (1.00 ~ 1.01) | |
| Widowed | 13 (3.93) | 1.00 (0.98 ~ 1.02) | |
| **Alcohol history** | | | 0.050 |
| Yes | 22 (6.65) | 0.98 (0.96 ~ 1.00) | |
| No | 309 (93.35) | 1.00 (1.00 ~ 1.01) | |
| **Smoking history** | | | 0.515 |
| Yes | 50 (15.11) | 1.00 (0.99 ~ 1.01) | |
| No | 281 (84.89) | 1.00 (1.00 ~ 1.01) | |
| **Medical history** | | | 0.768 |
| None | 48 (14.50) | 1.00 (0.99 ~ 1.01) | |
| Hypertension | 172 (51.96) | 1.00 (0.99 ~ 1.01) | |
| Diabetes | 75 (22.66) | 1.00 (0.99 ~ 1.01) | |
| Hypertension + Diabetes | 36 (10.88) | 1.01 (0.99 ~ 1.02) | |

| Variables | n (%) | OR (95%CI) | P for interaction |
|---|---|---|---|
| **Family history** | | | 0.214 |
| Yes | 50 (15.15) | 0.99 (0.98 ~ 1.01) | |
| No | 280 (84.85) | 1.00 (1.00 ~ 1.01) | |
| **Medication** | | | 0.174 |
| Typical antipsychotics | 40 (12.08) | 0.99 (0.98 ~ 1.00) | |
| Atypical antipsychotics | 229 (69.18) | 1.00 (1.00 ~ 1.01) | |
| Combination therapy | 62 (18.73) | 1.00 (1.00 ~ 1.01) | |
| **Chlorpromazine equivalent dose** | | | 0.821 |
| <300 mg/day | 156 (47.13) | 1.00 (1.00 ~ 1.01) | |
| 300–600 mg/day | 163 (49.24) | 1.00 (1.00 ~ 1.01) | |
| >600 mg/day | 12 (3.63) | 1.00 (0.98 ~ 1.02) | |
| **Age group** | | | 0.619 |
| 18–44 years | 48 (14.50) | 1.01 (0.99 ~ 1.02) | |
| 45–60 years | 125 (37.76) | 1.00 (0.99 ~ 1.01) | |
| 61–75 years | 158 (47.73) | 1.00 (1.00 ~ 1.01) | |
| **Disease duration** | | | 0.140 |
| <5 years | 9 (2.72) | 0.99 (0.96 ~ 1.02) | |
| 5–10 years | 15 (4.53) | 1.07 (0.99 ~ 1.15) | |
| 11–20 years | 53 (16.01) | 1.00 (0.99 ~ 1.01) | |
| >20 years | 254 (76.74) | 1.00 (1.00 ~ 1.01) | |
| **Serum uric acid** | | | 0.071 |
| Normal | 268 (80.97) | 1.00 (1.00 ~ 1.01) | |
| Hyperuricemia | 63 (19.03) | 0.99 (0.98 ~ 1.00) | |
| **BMI category** | | | 0.559 |
| Underweight | 14 (4.23) | 1.00 (0.98 ~ 1.02) | |

| Variables | n (%) | OR (95%CI) | P for interaction |
|---|---|---|---|
| Normal | 135 (40.79) | 1.00 (1.00 ~ 1.01) | |
| Overweight | 182 (54.98) | 1.00 (1.00 ~ 1.01) | |

OR: Odds Ratio, CI: Confidence Interval
